# N-fertilization and disturbance exert long-lasting complex legacies on subarctic ecosystems

**DOI:** 10.1007/s00442-024-05524-z

**Published:** 2024-03-13

**Authors:** Outi H. Manninen, Eero Myrsky, Anne Tolvanen, Sari Stark

**Affiliations:** 1 Arctic Centre, University of Lapland, Pohjoisranta 4, 96100 Rovaniemi, Finland; 2 Natural Resource Institute Finland, Paavo Havaksen Tie 3, 90570 Oulu, Finland

**Keywords:** Treeline ecotone, Plant recovery, Functional types, Microbial nitrogen, Vegetation change

## Abstract

Subarctic ecosystems are subjected to increasing nitrogen (N) enrichment and disturbances that induce particularly strong effects on plant communities when occurring in combination. There is little experimental evidence on the longevity of these effects. We applied N-fertilization (40 kg urea-N ha^−1^ year^−1^ for 4 years) and disturbance (removal of vegetation and organic soil layer on one occasion) in two plant communities in a subarctic forest-tundra ecotone in northern Finland. Within the first four years, N-fertilization and disturbance increased the share of deciduous dwarf shrubs and graminoids at the expense of evergreen dwarf shrubs. Individual treatments intensified the other’s effect resulting in the strongest increase in graminoids under combined N-fertilization and disturbance. The re-analysis of the plant communities 15 years after cessation of N-fertilization showed an even higher share of graminoids. 18 years after disturbance, the total vascular plant abundance was still substantially lower and the share of graminoids higher. At the same point, the plant community composition was the same under disturbance as under combined N-fertilization and disturbance, indicating that multiple perturbations no longer reinforced the other’s effect. Yet, complex interactions between N-fertilization and disturbance were still detected in the soil. We found higher organic N under disturbance and lower microbial N under combined N-fertilization and disturbance, which suggests a lower bioavailability of N sources for soil microorganisms. Our findings support that the effects of enhanced nutrients and disturbance on subarctic vegetation persist over decadal timescales. However, they also highlight the complexity of plant–soil interactions that drive subarctic ecosystem responses to multiple perturbations across varying timescales.

**Supplementary Information:**

The online version contains supplementary material available at 10.1007/s00442-024-05524-z.

## Introduction

Enhanced soil nutrient availability induced by climate warming (Jiang et al. 2016; McLaren and Buckeridge 2021; Pold et al. 2021; Salazar et al. 2020), atmospheric nitrogen (N) deposition (Choudhary et al. 2016), and human-induced disturbances (Wang and Friedl 2019) are increasingly modifying subarctic ecosystems. These anthropogenic perturbations occur in combination with natural disturbances such as herbivory (Jepsen et al. 2013; Sundqvist et al. 2019; Tuomi et al. 2021). Multiple perturbations induce more complex effects than what can be predicted based on single factors (Houseman et al. 2008; Tylianakis et al. 2008; Wilson and Tilman 1991); yet, a few experimental studies exist that consider the individual and the combined effects of perturbations on subarctic plant communities (Aerts 2010). Further, a largely unresolved question is whether these effects persist in the long-term, or whether subarctic plant communities may return to their initial state after perturbations cease, determining whether currently ongoing changes in vegetation are irreversible, or may be reversed (Gonzales et al. 2021; Liu et al. 2020; Speed et al. 2010; Werner et al. 2021).

The reason for the complex effects of multiple perturbations derives from the variation in plant species’ adaptations to soil nutrient regimes and the loss of biomass. Due to the strong nutrient limitation of subarctic ecosystems (Schimel and Bennett 2004), plant communities are very sensitive to increased N availability that lead to strong changes in community structure and productivity, and specifically, a higher share of fast-growing graminoids and deciduous dwarf shrubs (Grellmann 2002; Mack et al. 2004; Ylänne et al. 2020). Increased N may benefit fast-growing plant functional types which allocate surplus nutrients effectively for new growth, in contrast to slow-growing plant functional types such as evergreen dwarf shrubs which exhibit more conservative N use strategies (Eckstein and Karlsson 1997). Plant functional types are also characterized by their responses to disturbances. Graminoids and deciduous dwarf shrubs exhibit faster growth and recover more rapidly from the loss of biomass than slow-growing evergreen dwarf shrubs, which are more sensitive to physical damage (Aerts 2010; Olofsson et al. 2005; Tolvanen 1994; Tybirk et al. 2000). Consequently, enhanced nutrients commonly have synergistic effects with disturbances leading to especially strong shifts in vegetation when these two perturbations occur in combination (Manninen et al. 2011; Manninen and Tolvanen 2013; Strengbom and Nordin 2012). As plant species and functional types differ in their responses to N-addition and disturbances, the initial plant community structure is a major determinant for plant community responses to perturbations. In the subarctic region, habitats that are initially dominated by fast-growing plant functional types usually show a stronger response to nutrient addition (Gough et al. 2000; Pennings et al. 2005; Speed et al. 2010; Sundqvist et al. 2014) and recover more rapidly after disturbances (Olofsson et al. 2005; Sundqvist et al. 2020; Ylisirniö and Allén 2016) than habitats dominated by slow-growing plant functional types.

In principle, the long-term development of plant community composition and abundance after N-fertilization and disturbance could have two alternative trajectories. In a possible first trajectory, the slow-growing plant functional types eventually catch up with the fast-growing plant functional types, and return to the ecosystem by seed dispersal or the clonal growth of the dominant plant species from surrounding vegetation (Hautala et al. 2008; Olofsson et al. 2005). Many slow-growing evergreens, such as the mountain crowberry (*Empetrum nigrum* ssp. *hermaphroditum*) ((Hagerup) Böcher), possess a strong ability to outcompete other species (Bråthen et al. 2010, 2017; van Wijk et al. 2003) and would therefore be expected to return to the system in the long run if enough time is permitted. In a second possible trajectory, the post-perturbation plant community persists in the vegetation due to the indirect impacts of plant species composition on soil nutrient cycling (Hobbie 1992, 2015). As graminoids produce rapidly decomposing litter, their increased abundance maintains higher rates of nutrient cycling (De Deyn et al. 2008; Egelkraut et al. 2018a, b; Olofsson and Oksanen 2002) and creates a positive feedback further promoting their own growth through a more effective nutrient use (Chapin and Shaver 1989; Olofsson 2006; Ylänne et al. 2020). Studies from subarctic tundra ecosystems have indicated that graminoid-dominated vegetation induced by nutrient enrichment and disturbance may persist for a century even after the perturbations have ceased (Egelkraut et al. 2018a; Stark et al. 2019; Tømmervik et al. 2010). Combined nutrient enrichment and disturbance may also shift initially differing habitats into a similar type of graminoid-dominated habitat, indicating that this vegetation change may be relatively insensitive to the initial plant community composition (Egelkraut et al. 2018a).

Here, we tested the effects of N-fertilization and disturbance on subarctic vegetation composition over a time period of 18 years in the forest-tundra ecotone in Finnish Lapland. We implemented full factorial treatments of N-fertilization (*i.e.*, annual urea addition) and disturbance (*i.e.*, the total removal of the vegetation cover and organic soil layer) in two plant communities along an altitudinal gradient: a mountain birch (*Betula pubescens* ssp. *czerepanovii*) forest and a tundra. In agreement with earlier studies (Grellmann 2002; Mack et al. 2004; Ylänne et al. 2020), within the initial 4-year timeframe, both N-fertilization and disturbance alone increased the share of deciduous dwarf shrubs and graminoids at the expense of evergreen dwarf shrubs, and the strongest increase in graminoid share was detected under combined N-fertilization and disturbance (Manninen and Tolvanen 2013). The total plant abundance recovered more rapidly in the mountain birch forest with a higher share of graminoids and deciduous dwarf shrubs than in the tundra with a higher share of evergreen dwarf shrubs (Manninen and Tolvanen 2013), thus supporting earlier results that the initial community composition is important for plant community responses to perturbations (Speed et al. 2010; Sundqvist et al. 2014).

We analyzed vegetation composition and soil organic matter N stocks 15 years after ceasing N-fertilization and 18 years after disturbance treatment to test which of the alternative hypotheses would be supported. According to the first hypothesis (H1), the slow-growing dwarf shrubs catch up with the more rapid-growing plant functional types and return after perturbations (Aerts 2010; Gonzales et al. 2021; Olofsson et al. 2005). As plant communities with a higher share of rapidly growing plant functional types recover from perturbations at a faster rate than plant communities with a higher share of slow-growing plant functional types (Speed et al. 2010; Sundqvist et al. 2014), we expected the total plant abundance to be closer to the control level in the mountain birch forest than in the tundra. According to the second hypothesis (H2), an increased share of graminoids in the vegetation persists after perturbations (Liu et al. 2020; Olofsson 2006; Werner et al. 2021), and therefore, the long-term effects of perturbations on vegetation should not significantly differ from the short-term effects irrespective of the initial plant community composition (Egelkraut et al. 2018a). The share of graminoids should thus still be higher under N-fertilization and disturbance, and the greatest under combined N-fertilization and disturbance (Manninen and Tolvanen 2013).

## Materials and methods

### Study area and experimental design

The experiment was established in the Ounastunturi fell region in Hetta, Finnish Lapland (68°14′ N, 23°45′ E), in 2002 (Manninen and Tolvanen 2013). The mean annual temperature in the area is  – 0.6 °C and mean annual precipitation 532 mm (1991–2020) (Jokinen et al. 2021). The tops of the smooth-shaped Ounastunturi fells are on average 720 a.s.l. The mountain birch zone, dominated by *Betula pubescens* ssp. *czerepanovii,* reaches up to 455 a.s.l., above which is treeless tundra. The dominant species in the field layer are deciduous dwarf shrub *Vaccinium myrtillus* L. and evergreen dwarf shrubs *V. vitis-idaea* L. and *Empetrum nigrum* subsp. *hermaphroditum,* along with a lower abundance of graminoids such as *Deschampsia flexuosa* (L.) Trin. At the ground layer, moss *Pleurozium schreberi* L. and lichens *Cladonia* spp. are abundant.

The study area is inside the area of reindeer management in Finland, where reindeer graze freely over the landscape. Contrasting with many other countries with reindeer husbandry, seasonal pasture rotation in Finland is not always practiced (Stark et al. 2023). Outakka fell is located in the Näkkälä reindeer herding co-operative, which has a total area of 3,557 km^2^, and is grazed both during winter and summer (TOKAT data, Finnish Reindeer Herders Association). When the experiment was started in 2002, the average reindeer density during the preceding decade had been 1.8 reindeer/km^2^, rising to 2.1 reindeer/km^2^ between the years 2002–2005, and 2.8 reindeer/km^2^ between the years 2006–2018 with the highest density occurring between 2012 and 2013 with 3.9 reindeer/km^2^ (Reindeer Herders Association Statistics). Other herbivores include hare (*Lepus timidus*) and small rodents such as gray-sided voles (*Myodes rufocanus*), but the study area has not experienced the moth outbreaks which have been common in many other parts of northern Fennoscandia (Jepsen et al. 2008).

In 2002, we designed a full factorial experiment with two sites (mountain birch forest and treeless tundra heath at 450 and 540 m a.s.l., respectively), disturbance (undisturbed, disturbed), and N-fertilization (unfertilized, N-fertilized). At both sites, 28 permanent study plots, 0.5 m × 0.5 m in size, approximately 5 m apart, were established randomly in early June 2002 (*N* = 7 per treatment). The disturbance treatment was implemented by removing the vegetation and organic soil layers (approximately 4 cm in the mountain birch forest and 2 cm in the tundra heath) while leaving the mineral soil layer intact. This disturbance treatment resembles several anthropogenic disturbances that can occur at a range of spatial scales, such as pedestrian trampling, motor vehicle tracks, infrastructure building (Forbes et al. 2001), trampling by the reindeer along pasture rotation fences (Kumpula et al. 2011) or enclosures (Stark et al. 2022). We noted that a moss necromass formed a thick layer above the organic soil layer in the mountain birch forest, whereas this layer was lacking in the tundra heath. The fertilization treatment was implemented by applying urea-N-46% in granular form (corresponding to 40 kg N ha^−1^ year^−1^) to the plots, including a 15 cm buffer zone around the plots. The quantity of added N was selected based on other fertilization experiments, which enabled comparing our results to other experiments (Eskelinen 2010; Mack et al. 2004; van Wijk et al. 2003). N-fertilization treatment was followed by immediate watering with circa 4L of river water, and as water nutrient concentrations are very low in Finnish Lapland (Niemi 2010), the watering likely delivered insignificant amounts of nutrients. The N-fertilization treatment was repeated in early June during 2002, 2003, 2004, and 2005. The N-fertilization level is substantial when compared with average N deposition in northern Finland, which was estimated at 1–3 N ha^−1^ year^−1^ during the time of the active implementation of experiment (Leinonen 2001). During 2002–5, we analysed plant abundances using point-frequency method during the months of July–August using a point-frequency frame sized 0.5 m × 0.5 m with 100 line intersections (Manninen and Tolvanen 2013).

### Vegetation and soil analyses in 2020

We estimated vegetation composition using a point intercept frame with 10 pins and moved the frame five times, resulting in 50 hits. Due to the vertical growth and multilayer structure of vascular plants in the field layer, we recorded all intercepts of all plant species and pooled them to measure plant abundances at functional type or total vegetation levels. This corresponds roughly to their biomass (Jonasson 1988). The conversion factors to calculate biomass from point-frequency data vary among the plant functional types and the results thus do not directly depict biomass (Bråthen and Hagberg 2004). Mosses and lichens which grow in a single layer were recorded only once per intersect, and their measured abundance therefore corresponds to their cover at the ground layer. We grouped the individual plant species into the following plant functional types: (1) deciduous dwarf shrubs, (2) evergreen dwarf shrubs, (3) graminoids, and (4) forbs at the field layer. We calculated the relative proportion of the different plant functional types from the total vascular plant abundance for all years. *Betula nana* was present only in few plots and hits on *B. nana* were originated from plants rooted outside the plot boundaries and hence were removed from the data. The ground layer covers were classified into (1) mosses, (2) lichens, (3) litter, and (4) bare ground.

We took multiple soil samples (5 soil cores, diameter 2.5 cm) within each plot at a 5 cm depth, which were then pooled to form one composite sample per plot. The thicknesses of organic and mineral layers were recorded during sampling, but we did not separate soil organic and mineral layers in the samples due to the very thin organic layer in the disturbed plots. Samples were homogenized (mesh size, 2 mm) and stored at + 4 °C before analyses that were finished within 3–4 days of sampling. Samples were analyzed for soil moisture % (12 h, 105 °C; calculated as a percentage of soil fresh weight) and soil organic matter content (OMC%, loss on ignition, 475 °C, 4 h; calculated as a percentage of soil dry weight). The sampling area and the total sample weight were used to calculate soil organic matter (SOM) stock per area. Soil pH in the surface organic soil layer was measured in 3:5 v/v soil: water suspensions using distilled water, shaking, and leaving samples stand overnight before measuring soil pH (Denver Instrument Model 220). A sub-sample of ~ 4 g fresh soil was extracted for 2 h with 50 mL of 0.5 M K_2_SO_4_. Dissolved organic carbon (DOC) concentrations were analyzed with TOC-VCPH/N Total Organic Carbon Analyzer (Shimadzu Corporation, Kyoto, Japan). NH_4_–N and NO_3_–N concentrations were analyzed via flow injection analysis (Quickchem 8000 FIA Analyzer, A83200, Zellweger Analytics, USA). The total extractable N was determined by oxidizing all extractable N to NO3–N with potassium persulfate in 120 °C and analyzed as above. Extractable organic N was calculated by subtracting inorganic N concentrations from the total. Microbial C and N was extracted from the samples using 0.5 M K_2_SO_4_ after chloroform fumigation for 18 h (Brookes et al. 1985), and then analyzed as total extractable N and DOC. Microbial C and N were calculated from the difference between unfumigated and fumigated extractions.

### Statistical data analysis

We conducted four sets of statistical analyses. To test how vegetation had changed over time, we analyzed the effects of habitat (mountain birch forest, tundra), year, N-fertilization and disturbance, and their interactions, on the shares of plant functional types using a linear mixed model with treatments, habitat, and year as fixed factors and plot as a random factor. Year was assigned as a repeated factor with the plot as the subject. We tested only shares, because the point-frequency method used in 2020 deviated from that used in 2002–5. Parameters were tested using restricted maximum likelihood (REML). Due to the time gap between last measurements (2005 and 2020), we treated the repeated effect (year) as a categorical variable and used unstructured covariance structure in our models. The unstructured covariance structure allows different correlations for each measurement, without imposing a specific pattern on the correlations between measurements, thus providing flexibility to account for various variations between years. Using unstructured covariance structure was also confirmed by lowest AIC values compared to the other covariance structures. We removed non-significant four-way interaction from the full model at a significance level of *p* < 0.05 and, based on AIC comparison (Supplementary Table 1), and show results without the four-way interaction. As forbs were absent in the tundra, the treatment effects on their share over time were tested only in the mountain birch forest. In turn, as lichens were absent from the mountain birch forest, their share was tested only in the tundra. To meet the assumptions of the linear mixed model, logarithmic transformations were used to all plant functional types.

We tested the effects of treatments on the abundances of plant functional types in the field layer, and the ground layer covers in 2020 individually. Due to data distributions, we used a generalized linear model using habitat, N-fertilization and disturbance, and their interactions, in the full model as factors. For the abundance of deciduous dwarf shrubs, graminoids, and forbs, we applied the gamma distribution with a log-function, and for evergreen dwarf shrubs, we applied negative binomial with a log-function as the probability and link-function, respectively. We analysed soil data from 2020 (OMC%, SOM stock, pH, moisture%, C and N concentrations, and C and N stocks) by a linear model using habitat, N-fertilization and disturbance, and their interactions, as factors. Logarithmic transformations were used as necessary to meet the assumptions of the linear model. We carried out these analyses using SPSS Statistics, version 28 (IBM Corp. 2021).

Finally, we used the non-metric multidimensional scaling (NMDS) on plant abundances in 2002, 2005, and 2020 to visualize and investigate the changes in vegetation community composition, using the function “metaMDS” in package “vegan” for R (Oksanen et al. 2020). As our aim was to concentrate on the overall patterns of common plant species, we omitted plant species with a frequency of ≤ 2 as rare species can introduce noise in the NMDS-ordination before the analysis (Poos and Jackson 2012). We applied the analysis to a Bray–Curtis dissimilarity matrix, and remained with two-dimensional solutions where minimum stress values were less than 0.2. We also examined the correlation between individual plant species (in 2002, 2005, and 2020) and soil properties (in 2020) and NMDS-ordination pattern using the function “envfit” in the package “vegan” and produced ordination graphs with significant correlations at level *p* ≤ 0.05 with NMDS-ordination space. We also fitted ellipses defined by the 95% confidence interval around centroids based on site dissimilarity scores derived from the NMDS to differentiate treatments (control, N-fertilized, disturbed, N-fertilized, and disturbed) between habitats (mountain birch forest, tundra) in 2002, 2005, and 2020. NMDS-ordinations were performed using R version 3.6.2.

## Results

### The shares of plant functional types over the 18-year time period (2002–5, 2020)

**Fig. 1:**
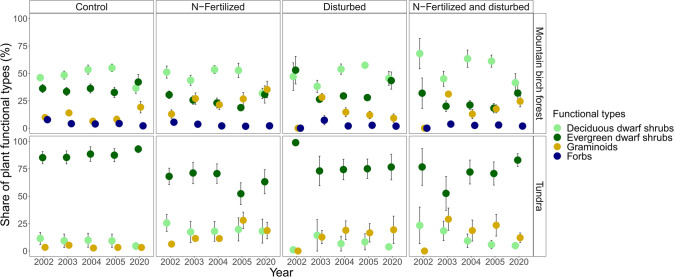
The shares of plant functional types (% of total abundance) within the treatments at two habitats in 2002–2005 and 2020

Over the entire 18-year period, mountain birch forest was characterized by a higher share of deciduous dwarf shrubs and graminoids compared to the tundra, while the tundra exhibited a higher share of evergreen dwarf shrubs (main effect of habitat, Table 1; Fig. 1).

**Table 1:** The effects of year, habitat, N-fertilization and disturbance, and their interactions on the relative proportions of plant functional types. F and *P *values are obtained by a linear mixed model

Source	Deciduous		Evergreen		Graminoids		Forbs*	
	F	*P*	F	*P*	F	*P*	F	*P*
Year	4.6	0.008	10.3	< .001	8.6	< .001	4.7	0.036
Habitat	38.9	< .001	150.6	< .001	6.3	0.016	na	na
N-fertilization	4.0	0.061	16.3	< .001	12.4	0.001	2.6	0.113
Disturbance	0.8	0.385	0.1	0.870	17.3	< .001	4.6	0.033
Year × Hab	1.6	0.204	2.6	0.045	2.5	0.058	na	na
Year × Fert	4.4	0.011	0.9	0.477	3.6	0.015	1.6	0.265
Year × Dist	1.6	0.214	3.6	0.013	3.0	0.042	1.4	0.337
Hab × Fert	3.3	0.085	1.6	0.208	1.3	0.252	na	na
Hab × Dist	0.2	0.649	0.3	0.560	10.9	0.002	na	na
Fert × Dist	0.2	0.639	0.9	0.358	17.9	< .001	0.0	0.982
Year × Hab × Fert	2.0	0.132	0.4	0.807	0.6	0.67	na	na
Year × Hab × Dist	3.7	0.020	0.9	0.468	0.8	0.488	na	na
Year × Fert × Dist	0.4	0.778	0.9	0.467	0.9	0.456	3.5	0.100
Hab × Fert × Dist	0.5	0.495	2.7	0.107	1.7	0.204	na	na

Logarithmic transformations were used to meet the requirements of the linear mixed model

^*^Tested only for the mountain birch forest

The share of deciduous shrubs decreased during the experiment (main effect of year, Table 1; Fig. 1). N-fertilization increased the share of deciduous shrubs during the initial years of the experiment, but this effect evened out in the long term (interaction effect of year and N-fertilization, Table 1; Fig. 1). The share of deciduous dwarf shrubs in the mountain birch forest decreased after disturbance only shortly after the treatment and increased gradually to the earlier level during the following years (interaction effect of year, habitat, and disturbance, Table 1; Fig. 1). On the contrary, in the tundra, the share of deciduous dwarf shrubs reached its peak after disturbance in the second year after the treatment, but their share gradually declined over the following years (Fig. 1).

The share of evergreen dwarf shrubs decreased during the first 4-year period but increased in 2020 at both habitats (main effect of year, Table 1; Fig. 1), with a more prominent increase in the mountain birch forest than the tundra (interaction effect of year and habitat, Table 1; Fig. 1). N-fertilization as a single treatment decreased the share of evergreen dwarf shrubs over the entire study period (main effect of N-fertilization, Table 1; Fig. 1). The effect of disturbance on the share of evergreen shrubs varied over time, and the share of evergreen shrubs after disturbance reached the level of the plots without disturbance by the fourth year after disturbance (interaction effect of year and disturbance, Table 1; Fig. 1).

The share of graminoids increased over the entire period at both habitats (main effect of year, Table 1; Fig. 1). N-fertilization as a single effect increased the share of graminoids throughout the 18-year period (main effect of N-fertilization, Table 1; Fig. 1), and this effect intensified over time (interaction effect of year and N-fertilization, Table 1; Fig. 1). The effect of disturbance on the share of graminoids was dependent on the habitat (main effect of disturbance, interaction effect of habitat and disturbance; Table 1; Fig. 1) and it also fluctuated over the years (interaction effect of year and disturbance, Fig. 1): when disturbance was applied as a sole treatment, it only shortly increased the share of graminoids in the mountain birch but increased the share of graminoids in the tundra throughout the experiment. In turn, when N-fertilization was combined with disturbance, the share of graminoids showed an increasing trend (interaction effect of N-fertilization and disturbance, Table 1; Fig. 1). The share of forbs, which was tested only in the mountain birch forest, declined over the study period (main effect of year, Table 1; Fig. 1) and after disturbance (main effect of disturbance, Table 1; Fig. 1).

### The plant abundances 15 years after N-fertilization and 18 years after disturbance in 2020

**Fig. 2:**
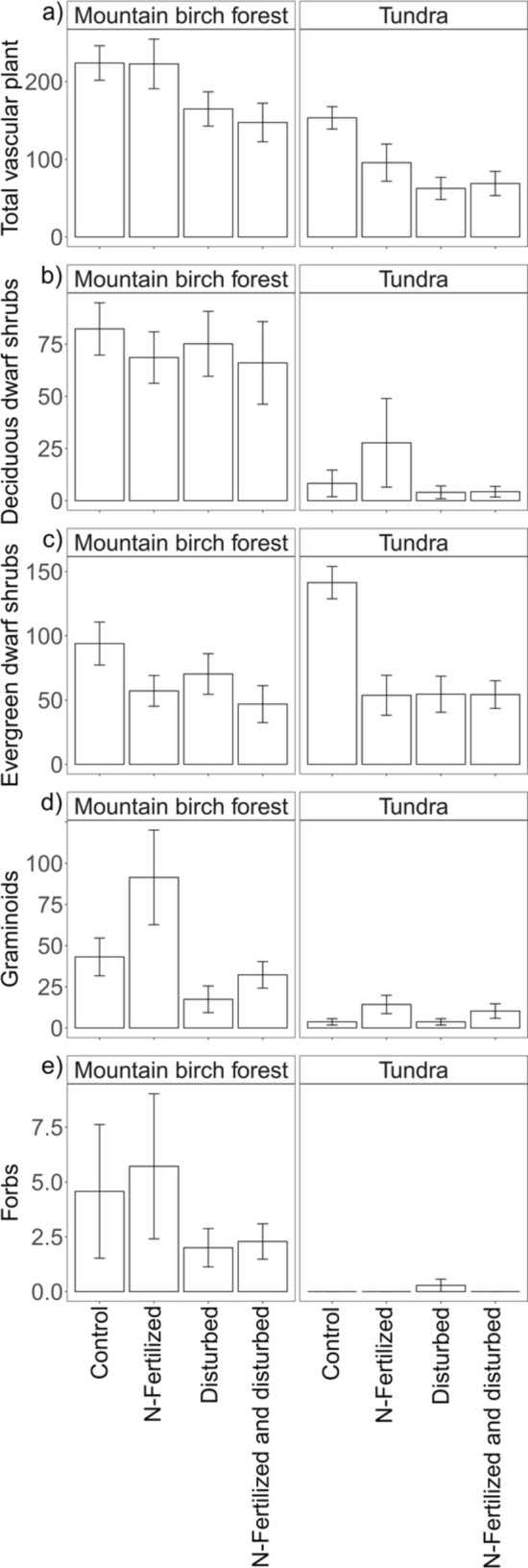
Plant abundances within the treatments at two habitats in 2020, 15, and 18 years after N-fertilization and disturbance, respectively. The panels present results of a point-frequency analyses performed for **a** total vascular plant abundance, **b** deciduous dwarf shrubs, **c** evergreen dwarf shrubs, **d** graminoids and **c** forbs, and expressed as hits per 100 pins. Values are mean ± SE

**Fig. 3:**
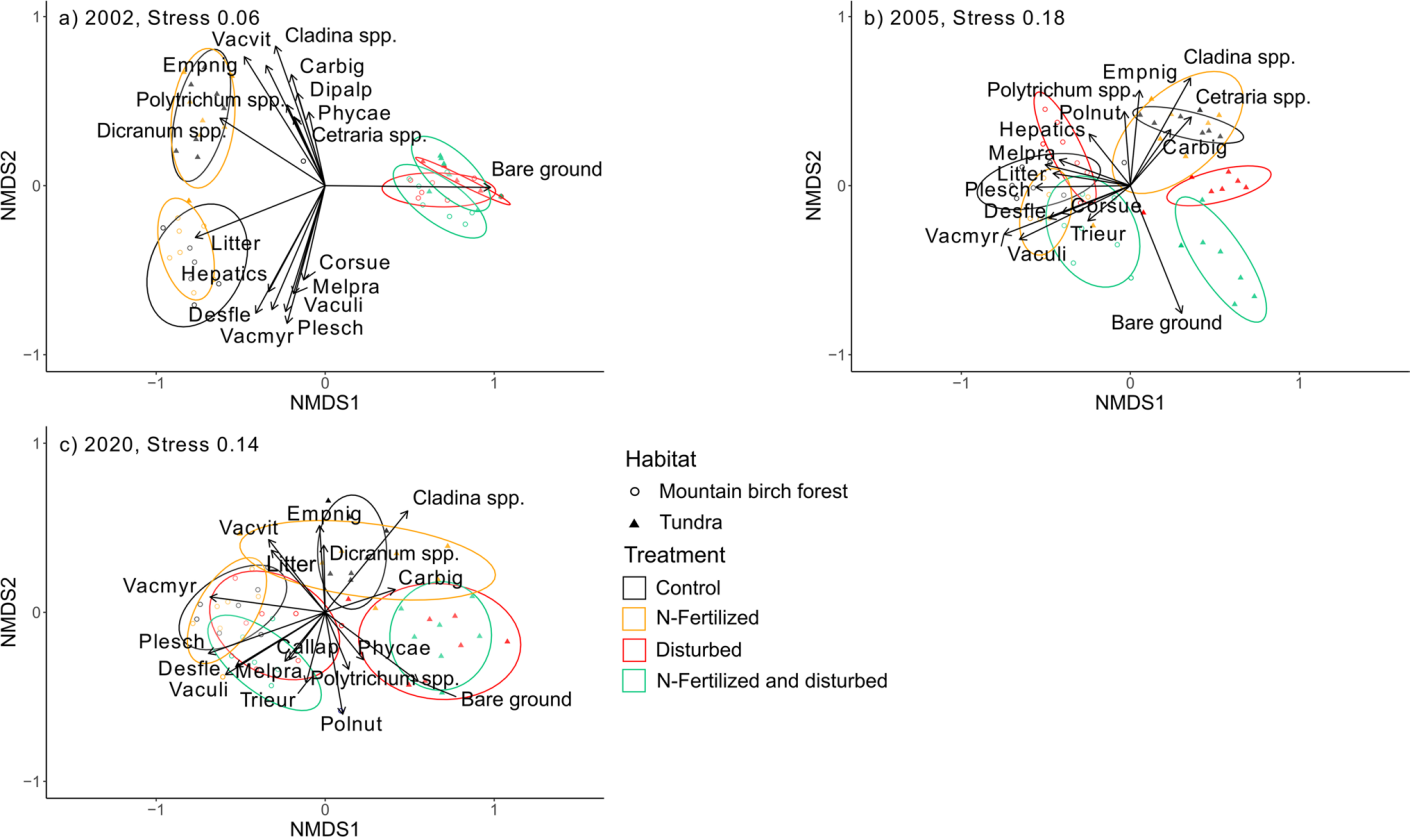
NMDS-ordination of plant species composition within treatments at two habitats in **a** 2002, after the treatments at the beginning of the experiment, **b** 2005, after 4 years of annual treatments of N-fertilization and disturbance, and **c** 2020, 15, and 18 years after N-fertilization and disturbance, respectively. Ellipses represent 95% confidence intervals for the treatments. Fit of the parameters are shown in Supplementary Tables 3, 4, 5

The mountain birch forest was characterized by higher total vascular plant, deciduous dwarf shrub, graminoid, and forb abundances than the tundra (main effect of habitat, Table 2; Fig. 3). The total vascular plant abundance was not affected by N-fertilization, but disturbance exerted a strong legacy: the total vascular plant abundance after disturbance was only 64% of that in the undisturbed plots irrespective of habitat or N-fertilization (main effect of disturbance, Table 2; Fig. 2a).

**Table 2:** The effects of habitat, N-fertilization and disturbance, and their interactions on the total vascular plant abundance and plant functional types

Source	Total vascular plant		Deciduous		Evergreen		Graminoids		Forbs*	
	F	*P*	F	*P*	F	P	F	P	F	*P*
Habitat	43.5	< 0.001	36.7	< 0.001	0.9	0.334	44.4	< 0.001	na	na
N-fertilization	1.5	0.223	0.5	0.477	16.1	< 0.001	8.1	0.004	0.3	0.593
Disturbance	19.3	< 0.001	6.5	0.011	10.6	0.001	3.0	0.086	8.3	0.004
Hab × Fert	0.3	0.567	1.9	0.172	0.6	0.453	1.1	0.298	na	na
Hab × Dist	0.1	0.769	5.2	0.022	2.0	0.157	1.9	0.164	na	na
Fert × Dist	0.7	0.406	0.7	0.402	7.5	0.006	0.9	0.347	0.1	0.779
Hab × Fert × Dist	2.0	0.162	0.9	0.343	4.0	0.045	0.4	0.539	na	na

F and *P* values are obtained by a generalized linear model

^*^Tested only for the mountain birch forest

The impact of disturbance on deciduous dwarf shrub abundance varied between habitats (interaction effect of habitat and disturbance, Table 2; Fig. 2b). 18 years after the disturbance treatment, in the mountain birch forest, the abundance of deciduous dwarf shrubs was almost at the same level as in plots without disturbance, whereas in the tundra, the abundance of deciduous dwarf shrubs was still about half of that in the plots without disturbance. We, however, note that this result may partially reflect the non-significant difference in deciduous dwarf shrub abundance after N-fertilization. Evergreen dwarf shrub abundance was substantially lower after both N-fertilization and disturbance (main effects of N-fertilization and Disturbance. Table 2; Fig. 2c). This negative effect was intensified after combined N-fertilization and disturbance, and to a stronger extent in the tundra than the mountain birch forest (interaction effects of N-fertilization and disturbance, and habitat, N-fertilization and disturbance, Table 2; Fig. 2c).

Graminoid abundance was substantially higher after N-fertilization irrespective of habitat or disturbance (main effect of N-fertilization, Table 2; Fig. 2d). Forbs, which were only present in the mountain birch forest, did not respond to N-fertilization, but their abundance in the disturbed plots was less than half of that in the undisturbed plots (main effect of disturbance, Table 2; Fig. 2e).

Moss and litter cover were higher in the mountain birch forest than the tundra, whereas lichens showed the opposite trend with a negligible cover in the mountain birch forest (main effect of habitat, Supplementary Table 1 and 2, lichens not tested for the habitat effect). There were no legacies of N-fertilization in the ground layer, but moss and lichen cover were still significantly lower after disturbance (main effect of disturbance, Supplementary Tables 1 and 2). Litter cover after disturbance was less than half of that in in plots without disturbance in the tundra, but there was no effect in the mountain birch forest (main effect of disturbance, interaction effect of habitat and disturbance, Supplementary Tables 1 and 2). Bare ground was absent in the mountain birch forest but had still a higher level after disturbance in the tundra (main effect of disturbance, Supplementary Tables 1 and 2).

### The community composition in 2002, 2005, and 2020

The NMDS-ordination in 2002 arranged plots into three groups: the mountain birch forest (both control and N-fertilization), the tundra (both control and N-fertilization), and all disturbed plots (disturbance and combined N-fertilization and disturbance in both habitats, Fig. 3a). Of the field layer species, deciduous dwarf shrubs (*V. myrtillus V. uliginosum)*, graminoids (*D. flexuosa*), and forbs (*M. pratense, C. suecica)* were associated with the mountain birch forest, and evergreen dwarf shrubs (*V. vitis-idaea*, *E. nigrum* ssp. *hermaphroditum, P. caerulea*) with the tundra (Supplementary Table 3; Fig. 3a), thus well reflecting the difference in the dominant plant functional type between the habitats. Of the ground layer, litter cover, a moss species *P. schreberi* and hepatics were associated with the mountain birch forest, and mosses *Polytrichum* spp. and *Dicranum* spp., and lichens *Cladina* spp. and *Cetraria* spp. with the tundra.

In 2005, the different treatments were more distinctively separated than in 2002 (Fig. 3b). In the mountain birch forest, the different treatments partially overlapped, whereas in the tundra, disturbance and combined N-fertilization and disturbance treatments were clearly separated from each other and from the plots without disturbance (both control and N-fertilization). Bare ground was associated with combined N-fertilization and disturbance treatment in both habitats. Reflecting the impact of nutrient addition on deciduous dwarf shrubs, graminoids and forbs, *V. myrtillus* and *V. uliginosum* and *D. flexuosa* were associated with N-fertilization in the mountain birch forest, whereas the forb *T. europaea* was most clearly associated with the combined N-fertilization and disturbance treatment (Supplementary Table 4; Fig. 3b). Hepatics and *Pohlia nutans*, typical early successional bryophytes, were associated with disturbance in the mountain birch forest (Supplementary Table 4; Fig. 3b). *E. nigrum* ssp. *hermaphroditum*, *P. caerulea*, *C. bigelowii*, *Cladina* spp., and *Cetraria* spp.) were associated with control and N-fertilization treatments in the tundra (Fig. 3b).

In 2020, the different treatments were closer together than in 2005 (Fig. 3c). In the mountain birch forest, there was no separation between control and N-fertilization treatments, and both disturbance and combined N-fertilization and disturbance treatments spread widely over them. *V. myrtillus* and moss *P. schreberi* were associated with control and N-fertilization treatments and *V. uliginosum*, *D. flexuosa* and *C. lapponica, M. pratense* and *T. europea* with all plots with disturbance (both disturbance and combined N-fertilization and disturbance; Supplementary Table 5; Fig. 3c). In the tundra, N-fertilization was spread over the control plots, with *V. vitis-idaea* and litter associated with N-fertilization, and *Dicranum* spp. with control plots. Disturbance and combined N-fertilization and disturbance treatments overlapped with each other in the ordination space (Fig. 3c), although these treatments had formed distinct groups in 2005 (Fig. 3b). *P. caerulea* and bare ground were associated with disturbance in the tundra, whereas *P. nutans* and *Polytrichum* spp. were associated with disturbance with no clear separation by habitat (Supplementary Table 5; Fig. 3c).

### Soil properties and their correlations with plant community composition 15 years after N-fertilization and 18 years after disturbance in 2020

**Fig. 4:**
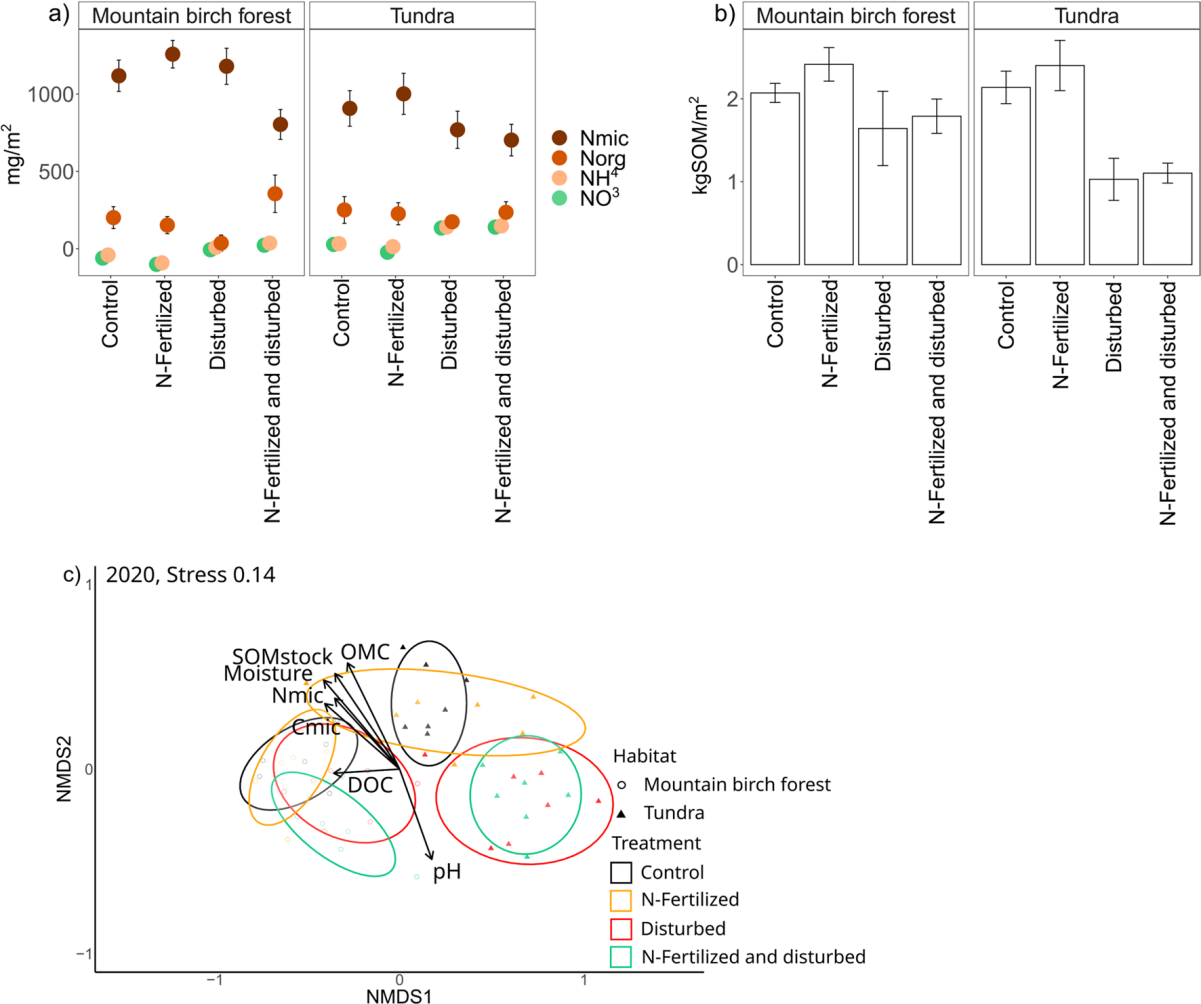
**a** Soil organic matter (SOM) stock, **b** soil and microbial N stocks within the treatments, values are mean + SE, and **c** NMDS-ordination describing associations of soil properties with treatments at two habitats in 2020. In (**c**), ellipses represent 95% confidence intervals for the treatments. Fit of the soil parameters are shown in Supplementary Table 8. Abbreviations are as follows: *NO*_*3*_*–N* nitrate–N, *NH*_*4*_*–N* ammonium–N, *N*_*org*_ organic N, *N*_*mic*_ microbial N, *C*_*mic*_ DOC dissolved organic C

OMC% and moisture% were lower and soil pH higher after disturbance (main effect of disturbance; Table 3 and 4). Moisture% was lower in the mountain birch forest than the tundra (main effect of habitat; Tables 3 and 4). The SOM stock had decreased in response to disturbance to a greater extent in the tundra than in the mountain birch forest (main effects of habitat and disturbance, interaction effect of habitat and disturbance; Table 5; Fig. 4a). N-fertilization had no effects on any of the analysed soil properties.

**Table 3:** The effects of habitat, N-fertilization, disturbance, and their interactions on OMC%, moisture%, SOM stock (g m^−2^), and soil pH

Source	OMC%*		Moisture%		SOM stock*		Soil pH	
	F	*P*	F	*P*	F	*P*	F	*P*
Habitat	1.6	0.207	5.0	0.030	5.7	0.021	0.0	0.868
N-fertilization	0.3	0.610	0.0	0.986	2.1	0.160	0.5	0.503
Disturbance	45.8	< 0.001	22.1	< 0.001	30.0	< 0.001	33.7	< 0.001
Hab × Fert	0.2	0.674	0.4	0.533	0.0	0.869	0.0	0.911
Hab × Dist	3.6	0.063	1.1	0.311	5.0	0.030	0.4	0.515
Fert × Dist	0.5	0.502	0.1	0.771	0.1	0.724	0.0	0.906
Hab × Fert × Dist	0.2	0.661	0.0	0.904	0.0	0.898	0.2	0.666

F and *P *values are obtained by a linear mixed model

^*^Logarithmic transformations were used to meet the requirements of the linear mixed model

**Table 4:** Soil properties and soluble and microbial N and C stocks per soil area in 2020

	Mountain birch forest				Tundra heath			
	Control	N-fertilized	Disturbed	N-fertilized and disturbed	Control	N-fertilized	Disturbed	N-fertilized and disturbed
OMC %	14.4 (1.8)	17.7 (3.8)	9.0 (4.7)	7.6 (1.4)	21.4 (5.1)	19.5 (6.2)	4.2 (1.4)	4.2 (0.5)
Moisture %	38. 7 (4.1)	40.0 (4.6)	28.2 (5.8)	30.3 (1.8)	37.4 (4.4)	34.5 (4.5)	20.6 (2.4)	20.0 (1.0)
Soil pH	4.29 (0.05)	4.30 (0.03)	4.58 (0.10)	4.63 (0.13)	4.22 (0.04)	4.31 (0.11)	4.65 (0.08)	4.66 (0.08)
DOC	4.07 (0.48)	4.26 (0.63)	4.20 (0.29)	5.30 (0.72)	3.60 (0.39)	3.28 (0.34)	2.66 (0.22)	3.51 (0.62)
Microbial C	12.3 (0.7)	13.6 (0.5)	12.2 (0.8)	13.4 (1.0)	9.2 (0.7)	11.0 (0.5)	9.2 (1.0)	8.7 (0.9)

*N* = 6 in the mountain birch forest, *N* = 7 in the tundra heath. Values are mean and S.E. in parentheses

**Table 5:** The effects of habitat, N-fertilization, disturbance, and their interactions on soil NH_4_–N, NO_3_–N, extractable organic N, microbial N. dissolved organic carbon (DOC), and microbial C stocks per area

	NH_4_–N *		NO_3_–N *		Organic N*		Microbial N		DOC		Microbial C	
	F	*P*	F	*P*	F	*P*	F	*P*	F	*P*	F	*P*
Habitat	1.2	0.284	3.4	0.072	1.1	0.304	20.3	< 0.001	12.3	0.001	37.0	< 0.001
N-fertilization	1.2	0.281	0.3	0.608	2.7	0.109	0.2	0.639	1.8	0.192	2.8	0.099
Disturbance	0.9	0.361	9.0	0.004	9.1	0.004	15.9	< 0.001	0.1	0.736	1.4	0.237
Hab × Fert	0.9	0.356	0.0	0.913	0.6	0.447	0.9	0.346	0.3	0.582	0.2	0.629
Hab × Dist	3.3	0.078	0.2	0.678	0.0	0.970	0.2	0.669	1.9	0.176	0.9	0.359
Fert × Dist	1.7	0.200	0.0	0.970	1.7	0.198	6.4	0.015	2.3	0.137	1.3	0.262
Hab × Fert × Dist	0.9	0.351	0.2	0.637	1.5	0.221	1.5	0.227	0.0	0.853	1.1	0.299

F and *P* values were obtained by a linear mixed model

^*^Logarithmic transformations were used to meet the assumptions of the linear mixed model

Microbial N and C as well as DOC stocks were higher in the mountain birch forest than the tundra (main effect of habitat, Tables 3 and 4, Fig. 4b). NO_3_–N stock was higher and organic and microbial N stocks lower after disturbance, but there were no effects of N-fertilization (Table 5; Fig. 4b). However, an interactive effect of N-fertilization and disturbance was found: disturbance decreased microbial N in the N-fertilized plots, when it had no effect in the unfertilized plots (main effects and interaction effect of disturbance and N-fertilization, Table 5; Fig. 4b). The concentrations of NH_4_–N, NO_3_–N, microbial N, DOC, and microbial C per g SOM were higher after disturbance (main effect of disturbance; Supplementary Tables 6 and 7), and in the case of NO_3_–N concentration, the effect was stronger in the tundra than the mountain birch forest (interaction effect of habitat and disturbance; Supplementary Tables 6 and 7). N-fertilization and disturbance affected microbial N concentration interactively (Supplementary Tables 6 and 7).

The correlation of all soil properties (OMC%, SOM stock, soil pH, and moisture %) with the NMDS-ordination space were statistically significant (Fig. 4c, Supplementary Table 8). OMC%, moisture % and SOM stock had the best fits with NMDS-ordination space and increased towards undisturbed treatment (both control and N-fertilization) without clear indication of habitat. Microbial N and C increased towards undisturbed plots, whereas DOC increased towards undisturbed plots in the mountain birch forest. The soil pH and NH_4_–N, NO_3_–N, microbial C, and DOC concentration correlated significantly with NMDS-ordination space (Supplementary Table 8), increasing towards disturbed plots without clear indication of habitat.

## Discussion

Here, we analyzed the legacies of N-fertilization and disturbance on vegetation both alone and in combination. Our aim was to test which of the two possible trajectories the long-term change in plant community composition and the abundances of plant functional types would follow: (H1) that, at decadal timescale, slow-growing plant functional types catch up with the fast-growing plant functional types resulting in the return of the original vegetation, or (H2) that graminoid-rich vegetation sustains over time. The plant community composition after the sole treatments of N-fertilization and disturbance largely supported H2. However, although at the short term, the strongest increase in graminoids was detected under combined N-fertilization and disturbance (Manninen and Tolvanen 2013), multiple perturbations no longer intensified the other’s effect, which supported neither H1 nor H2. Our findings highlight the persistency of vegetation change in response to N-fertilization and disturbances in subarctic ecosystems (Egelkraut et al. 2018a; Liu et al. 2020; Werner et al. 2021; Ylisirniö and Allén 2016), but further reveal that the cumulative impacts of multiple perturbations may be more transient over time.

In accordance with H2, graminoid abundance at the end of the experiment was still higher in N-fertilized than control plots 15 years after the treatments had ceased. Our study expands the timeframe of graminoid persistence even longer than earlier experimental studies that used the timeframe of 6–8 years after the cessation of NPK-fertilization (Liu et al. 2020; Werner et al. 2021). We used urea–N as fertilizer, which decomposes to NH_4_–N in the soil (Sinsabaugh et al. 2000), after which it is rapidly taken up by plants and immobilized by soil microorganisms (Barthelemy et al. 2018), or further oxidized to NO_3_–N through nitrification (Hartley et al. 1999; Schimel and Bennett 2004). Despite we used a highly soluble N source in the N-fertilization treatment, the share of graminoids actually increased over time, demonstrating that this change in vegetation was getting stronger over time even without continued nutrient addition. ^15^N-labeled urea addition experiments have demonstrated that Arctic vascular plants and bryophytes are very efficient in capturing and preserving the added N in their biomass (Barthelemy et al. 2018; Blok et al. 2016), and therefore, nutrients added through urea are efficiently maintained in the system.

Concurrently with an increased abundance of graminoids, evergreen dwarf shrub abundance was still substantially lower in plots that had received N-fertilization 15 years previously. *E. nigrum* ssp. *hermaphroditum*, which constituted 80% of evergreen dwarf shrubs, decreased during the first four years under annually repeated N-addition (Manninen and Tolvanen 2013). Earlier studies have associated the decline in evergreen dwarf shrubs under nutrient enrichment with shading by the more fast-growing plant species, reduced ericoid mycorrhizal ability to obtain recalcitrant nutrients (Bret-Harte et al. 2008; Gough et al. 2012), or increasing frequency of winter injuries (Street et al. 2015), whereas others have found more direct effects of fertilization (Wardle et al. 2013). Contrasting with the responses detected aboveground, we found no legacies of N-fertilization on soluble and microbial C and N stocks, which agrees with several earlier studies that found decoupled responses to fertilization between the above- and belowground (Jonasson et al. 1999; McLaren and Buckeridge 2019; Wardle et al. 2013). Some studies have found a long-term legacy of fertilization on nutrient pools and microbial biomass (Liu et al. 2020; Werner et al. 2021), whereas others have found weak effects as the added nutrients are efficiently taken up by the plant biomass (Hicks et al. 2020; Stark and Kytöviita 2006).

Agreeing with findings that vegetation recovery takes up several decades after disturbance (Heim et al. 2021; Ylisirniö and Allén 2016), the total vascular plant abundance was still lower 18 years after disturbance. In the NMDS-ordination, disturbance was associated with bare ground and early successional mosses such as *P. nutans* and *Polytrichium* spp. (Turetsky et al. 2012), demonstrating an early stage in the recovery process. By the year 2005, the recovery of the total vascular plant abundance after disturbance was lower in the tundra heath than the mountain birch forest due to the slow recovery of dominant evergreen dwarf shrubs (Manninen and Tolvanen 2013). In the long term, however, the total vascular plant recovery from disturbance no longer differed between the habitats, which supports H2. Slow return of the original vegetation after perturbations in subarctic ecosystems is associated with the same factors that limit plant growth, such as low temperatures and nutrient availability, and may also link with effects on soil moisture (Berner et al. 2020; Speed et al. 2010). Depicting the opportunistic growth strategy of graminoids (Hawkes and Sullivan 2001), the share of graminoids was higher in the disturbed plots, and—despite other plant functional types still showing a lower abundance—graminoids had returned to the control level at both habitats. Evergreen dwarf shrubs had also recovered at an equal rate in both habitats, and consistent with earlier studies (Aerts 2010; Bråthen et al. 2017; Olofsson et al. 2005), their recovery was lower compared to deciduous dwarf shrubs.

A novel finding of our study was that although short-term effects indicated particularly strong effects of parallel perturbations (Bret-Harte et al. 2008; Manninen et al. 2011; Manninen and Tolvanen 2013), without the annually repeated N additions, the combined N-fertilization treatment and the disturbance treatment no longer differed from each other. This was indicated both in the abundances of plant functional types and the NMDS-ordination, and supported neither H1 nor H2. As the single effect of N-fertilization on graminoids was highly persistent, this raises the question of which mechanism might explain why the initially strong synergistic effect of N-fertilization and disturbance disappeared without continuous N-input. Notably, this result did not correspond with findings from the belowground. As usual for subarctic ecosystems (Jonasson et al. 1999; McLaren and Buckeridge 2019; Stark et al. 2014), soil microbial N constituted the largest N stock that exceeded inorganic and soluble organic N stocks by several orders of magnitude, but—interestingly—it was the lowest under combined N-fertilization and disturbance. The leaching of nutrients from tundra ecosystems increases during periods when plants are not taking up nutrients (Treat et al. 2016), and therefore, the lower vegetation cover in the disturbed plots could have subjected the extra N to leaching. However, disturbance increased the K_2_SO_4_-extractable organic N, which does not support this assumption. Rather, soil organic N seemed less bioavailable for soil microorganisms after disturbance. A large proportion of the soil organic N is not directly utilizable due to, *e.g.*, precipitation of N-compounds with plant phenolics and an aggregation with mineral soil particles (Adamczyk et al. 2008; Eskelinen et al. 2009; Hättenschwiler and Vitousek 2000; Knicker 2011). High soil N concentrations promote organic compound stabilization through various chemical reactions and through modifying the degradation capacity of the soil microbial community (Bonner et al. 2022; Knicker 2011; Wei et al. 2023). The earlier vegetation under combined N-fertilization and disturbance could also still exert legacies on soil organic matter quality. In particular, graminoids are known to form dense fibrous mats containing high concentrations of decomposition-resistant aliphatic compounds that often accumulate in soils (De Deyn et al. 2008; Freschet et al. 2013; Rasse et al. 2005), which is reflected in the chemical composition of the soil organic matter (Eskelinen et al. 2009; Väisänen et al. 2015).

In addition to the plots that were previously subjected to the experimental treatments of N-fertilization and disturbance over an 18-year time period, the relative proportions of plant functional types had also changed in the control plots. We found an increasing trend in the relative proportion of graminoids and decreasing trends in the relative proportions of deciduous dwarf shrubs over time. These overall trends could result from both the climate warming and the intensity of herbivory in the area. Both deciduous and evergreen dwarf shrubs (particularly *E. nigrum* ssp. *hermaphroditum*) have increased substantially as a result of climate warming (Maliniemi et al. 2017; Vowles et al. 2017; Vuorinen et al. 2017), but warming also increases graminoids (Björkman et al. 2020), which means that climate warming may easily cause shifts in the relative proportions of different plant growth forms. Reindeer densities in the Näkkälä herding co-operative were relatively low before the initiation of the experiment if compared with more recent years. Physical damage through trampling (Egelkraut et al. 2020) and other herbivore-associated disturbances added by N-fertilization from urine and fecal deposition often result in an increase in grazing-tolerant graminoids and a decrease in deciduous dwarf shrubs (Eskelinen and Oksanen 2006; Sundqvist et al. 2019; Tuomi et al. 2019). An increased intensity of herbivory could therefore underlie the overall shifts in vegetation. This could also contribute to the persistence of graminoids after N-fertilization and disturbance, as herbivory is more intense in graminoid-rich vegetation patches than in the surrounding dwarf shrub tundra (Egelkraut et al. 2018a).

The high persistence of graminoids in the vegetation after N-fertilization and disturbance relates with the theory that graminoid-dominated tundra may represent an alternative, self-maintaining state of tundra vegetation (Van der Wal 2006). It has been suggested that a transition from undisturbed dwarf shrub—tundra to a disturbed graminoid—tundra represents an ecosystem state shift that, once taken place, is maintained through accelerated nutrient cycling rates that parallels increased plant productivity (Egelkraut et al. 2018a; Van der Wal 2006; Zimov et al. 1995). Whether these alternative states represent transient or stable states depends on the definition of stability in terms of timescale (Egelkraut et al. 2018b; Fukami 2015). Noteworthy, an ecosystem state shift is usually defined as a change in the dominant plant functional type (Falk et al. 2022; Van der Wal 2006), whereas both our study and earlier N-fertilization experiments from subarctic tundra (Liu et al. 2020; Werner et al. 2021) show that the higher share of graminoids persists in the vegetation for a long time even when dwarf shrubs remain dominant. We suggest that the distinction between alternative vegetation states may be more complicated and subtler than so far described. The longevity of vegetation change may also depend on the type of perturbation. A sparse graminoid vegetation interspersed with non-vegetated ground that results from disturbances may not form a functionally self-sustaining grassland, where dwarf shrubs cannot establish (sensu Egelkraut et al. 2018b). After disturbance, the plant community composition may thus depend on whether fast-growing plant functional types manage to cover the area before dwarf shrubs.

In summary, our experiment covering an 18-year timeframe showed persistent effects of N-fertilization and disturbance on subarctic plant communities, thus demonstrating high longevity of vegetation change in ecological timeframe and for the perspective of environmental management. Our findings add to previous evidence on the sensitivity of subarctic ecosystems to shift towards a novel vegetation state as a result of nutrient enrichment (Liu et al. 2020; Van der Wal 2006; Werner et al. 2021). They also confirm a slow recovery of vegetation after intense disturbances (Heim et al. 2021; Ylisirniö and Allén 2016). However, although N-fertilization and disturbance had reinforced each other’s effects in the short term (Manninen and Tolvanen 2013), this synergistic interaction of multiple perturbations vanished over time, most likely due to nutrient stabilization into chemical forms that were less available for plants and soil microorganisms. Complexities associated with climate warming and disturbances have been recognized as a major challenge that prevents the creation of accurate predictions for future vegetation change, as these trends appear spatially and temporally variable (Phoenix and Treharne 2022). Our long-term experiment provided mechanistic understanding on the factors that could underlie this variation. Counterintuitively, N-fertilization and disturbance alone exerted persistent effects on vegetation, but their combined effect was transient in the vegetation but persistent in the soil.

### Supplementary Information

Below is the link to the electronic supplementary material.

Supplementary file1 (DOCX 47 KB)

## Data Availability

The datasets used in this study are available from the corresponding author on reasonable request.
