# Supplementary material for: N-fertilization and disturbance exert long-lasting complex legacies on subarctic ecosystems
Source: Oecologia. 2024 Mar 13;204(3):689–704. doi: 10.1007/s00442-024-05524-z (PMC10980618; doi:10.1007/s00442-024-05524-z)
Supplement: Supplementary file 1 — Supplementary file1 (DOCX 47 KB) [file 442_2024_5524_MOESM1_ESM.docx]

**Supplemetary Table 1.** AIC values for the full model with four-way interaction (Year, Habitat, N-fertilization and Disturbance) and three-way interaction model for the share of plant functional types.

| Model | AIC value | deltaAIC |
| --- | --- | --- |
|  |  |  |
| 4-way interaction- model (full model) |  |  |
| Deciduous | -0.43 |  |
| Evergreen | -82.98 |  |
| Graminoids | 162.13 |  |
| Forbs | na |  |
| 3-way interaction- model |  |  |
| Deciduous | 1.54 | 1.97 |
| Evergreen | -87.31 | 4.32 |
| Graminoids | 163.82 | 1.69 |
| Forbs | 51.08 | na |
|  |  |  |

**Supplementary Table 2.** The effects of habitat, N-fertilization and disturbance, and their interactions on the cover of mosses, lichens and litter. F- and P-values are obtained by a generalized linear model.

| Source | Moss | | Lichens* | | Litter | | Bare ground* | |
| --- | --- | --- | --- | --- | --- | --- | --- | --- |
|  | F | *P* | F | *P* | F | *P* | F | *P* |
|  |  |  |  |  |  |  |  |  |
| Habitat | 51.4 | **<0.001** | na | na | 5.0 | **0.026** | na | na |
| Fertilization | 3.3 | 0.068 | 1.9 | 0.321 | 0.3 | 0.587 | 0.0 | 0.869 |
| Disturbance | 4.5 | **0.033** | 5.2 | **0.022** | 4.9 | **0.028** | 66.3 | **<0.001** |
| Hab × Fert | 1.7 | 0.193 | na | na | 0.1 | 0.797 | na | na |
| Hab × Dist | 0.1 | 0.721 | na | na | 14.0 | **<0.001** | na | na |
| Fert × Dist | 1.0 | 0.322 | 0.5 | 0.565 | 0.2 | 0.689 | 0.1 | 0.767 |
| Hab × Fert × Dist | 0.1 | 0.722 | na | na | 0.2 | 0.689 | na | na |

*Tested only for the tundra**.**

**Supplementary Table 3.** The cover of mosses, lichens, litter and bare ground in the ground layer in 2020. N = 6 in the mountain birch forest, N = 7 in the tundra. Values are mean and S.E. in parentheses.

|  | Mountain birch forest | | | | Tundra | | | |
| --- | --- | --- | --- | --- | --- | --- | --- | --- |
|  | Control | N-fertilized | Disturbed | N-fertilized and Disturbed | Control | N-fertilized | Disturbed | N-fertilized and Disturbed |
| Mosses | 57.1 (9.5) | 49.4 (8.9) | 42.3 (6.8) | 42.9 (7.1) | 25.7 (6.3) | 17.1 (3.9) | 18.6 (2.6) | 14.9 (2.8) |
| Lichens | 0.9 (0.6) | 0.0 (0.0) | 2.3 (1.3) | 2.3 (1.3) | 19.1 (3.7) | 24.9 (5.4) | 13.1 (1.4) | 14.0 (6.0) |
| Litter | 42.0 (9.4) | 50.0 (9.0) | 53.7 (6.2) | 53.7 (6.9) | 52.9 (7.1) | 54.3 (9.4) | 23.1 (5.3) | 24.6 (6.6) |
| Bare ground | 0.0 (0.0) | 0.0 (0.0) | 0.3 (0.3) | 1.1 (0.7) | 0.0 (0.0) | 2.0 (1.2) | 36.6 (7.5) | 36.0 (4.8) |

**Supplementary Table 4**. Correlations (r^2^) of plant species abundance in the field layer and the cover of ground layer with the NMDS-ordination pattern describing single and combined N-fertilization and disturbance treatments in 2002. Significance of correlations (P) is based on 999 permutations. Signif. codes: 0 '***' 0.001 '**' 0.01 '*' 0.05 '.' 0.1 ' ' 1

|  | r^2^ | Pr(>r) |
| --- | --- | --- |
|  |  |  |
| *Vaccinium myrtillus* | 0.7376 | 0.001 *** |
| *Vaccinium uliginosum* | 0.6089 | 0.001 *** |
| *Vaccinium vitis-idaea* | 0.6272 | 0.001 *** |
| *Empetrum nigrum* ssp. *hermaphroditum* | 0.8105 | 0.001 *** |
| *Phyllodoce caerulea* | 0.1985 | 0.001 *** |
| *Deschampsia flexuosa* | 0.6363 | 0.001 *** |
| *Carex bigelowii* | 0.4714 | 0.001 *** |
| *Melanpyrum pratense* | 0.4427 | 0.001 *** |
| *Cornus suecica* | 0.3274 | 0.001 *** |
| *Diphasiastrum alpinum* | 0.3258 | 0.001 *** |
| *Pleurozium schreberi* | 0.7142 | 0.001 *** |
| *Polytrichum* sp. | 0.2796 | 0.001 *** |
| *Dicranum* sp. | 0.5473 | 0.001 *** |
| *Cladina* sp. | 0.7671 | 0.001 *** |
| *Cetraria* sp. | 0.1992 | 0.003 ** |
| Hepatics | 0.5056 | 0.001 *** |
| Litter | 0.6846 | 0.001 *** |
| Bare ground | 0.9578 | 0.001 *** |
|  |  |  |

**Supplementary Table 5**. Correlations (r^2^) of plant species abundance in the field layer and the cover of ground layer with the NMDS-ordination pattern describing single and combined N-fertilization and disturbance treatments in 2005. Significance of correlations (P) is based on 999 permutations. Signif. codes: 0 '***' 0.001 '**' 0.01 '*' 0.05 '.' 0.1 ' ' 1

|  | r^2^ | Pr(>r) |
| --- | --- | --- |
|  |  |  |
| *Vaccinium myrtillus* | 0.6420 | 0.001 *** |
| *Vaccinium uliginosum* | 0.5315 | 0.001 *** |
| *Vaccinium vitis-idaea* | 0.0399 | 0.338 |
| *Empetrum nigrum* ssp. *hermaphroditum* | 0.3206 | 0.001 *** |
| *Phyllodoce caerulea* | 0.0912 | 0.047 * |
| *Deschampsia flexuosa* | 0.2694 | 0.002 ** |
| *Carex bigelowii* | 0.1669 | 0.004 ** |
| *Trientalis europea* | 0.1065 | 0.030 * |
| *Melanpyrum pratense* | 0.2692 | 0.001 *** |
| *Cornus suecica* | 0.1914 | 0.002 ** |
| *Lycopodium clavatum* | 0.0620 | 0.156 |
| *Pleurozium schreberi* | 0.3133 | 0.001 *** |
| *Polytrichum* sp*.* | 0.1932 | 0.008 ** |
| *Dicranum* sp. | 0.0880 | 0.078 . |
| *Pohlia nutans* | 0.1525 | 0.012 * |
| *Cladina* sp. | 0.5314 | 0.001 *** |
| *Cetraria* sp. | 0.2953 | 0.001 *** |
| Hepatics | 0.1998 | 0.003 ** |
| Litter | 0.2141 | 0.003 ** |
| Bare ground | 0.6620 | 0.001 *** |
|  |  |  |

**Supplementary Table 6.** Correlations (r^2^) of plant species abundance in the field layer and the cover of ground layer with the NMDS-ordination pattern describing single and combined N-fertilization and disturbance treatments in 2020. Significance of correlations (P) is based on 999 permutations. Signif. codes: 0 '***' 0.001 '**' 0.01 '*' 0.05 '.' 0.1 ' ' 1

|  | r^2^ | Pr(>r) |
| --- | --- | --- |
|  |  |  |
| *Vaccinium myrtillus* | 0.4722 | 0.001 *** |
| *Vaccinium uliginosum* | 0.4838 | 0.001 *** |
| *Vaccinium vitis-idaea* | 0.2967 | 0.001 *** |
| *Empetrum nigrum* ssp. *hermaphroditum* | 0.2644 | 0.001 *** |
| *Phyllodoce caerulea* | 0.1306 | 0.017 * |
| *Deschampsia flexuosa* | 0.3849 | 0.001 *** |
| *Calamagrostis lapponica* | 0.1229 | 0.022 * |
| *Carex bigelowii* | 0.1902 | 0.003 ** |
| *Trientalis europea* | 0.1876 | 0.003 ** |
| *Melanpyrum pratense* | 0.1402 | 0.009 ** |
| *Cornus suecica* | 0.0615 | 0.165 |
| *Pleurozium schreberi* | 0.5365 | 0.001 *** |
| *Polytrichum* sp. | 0.1328 | 0.020 * |
| *Dicranum* sp. | 0.1572 | 0.005 ** |
| *Pohlia nutans* | 0.3743 | 0.001 *** |
| *Cladina* sp. | 0.5981 | 0.001 *** |
| *Cetraria* sp. | 0.0857 | 0.092 . |
| Litter | 0.2360 | 0.001 *** |
| Bare ground | 0.4699 | 0.001 *** |
|  |  |  |

**Supplementary Table 7.** The effects of habitat, N-fertilization, disturbance, and their interactions on soil NH_4_-N, NO_3_-N, extractable organic N, microbial N, dissolved organic carbon (DOC) and microbial C concentrations per soil organic matter. F- and *P*- values obtained using linear mixed model.

|  | NH_4_-N* | | NO_3_-N* | | Organic N* | | Microbial N | | DOC | | Microbial C | |
| --- | --- | --- | --- | --- | --- | --- | --- | --- | --- | --- | --- | --- |
|  | F | *P* | F | *P* | F | *P* | F | *P* | F | *P* | F | *P* |
| Habitat | 0.2 | 0.650 | 0.0 | 0.954 | 0.7 | 0.415 | 4.0 | 0.053 | 0.0 | 0.863 | 0.0 | 0.951 |
| Fertilization | 0.1 | 0.826 | 0.0 | 0.843 | 2.2 | 0.143 | 8.0 | **0.007** | 0.1 | 0.812 | 1.5 | 0.231 |
| Disturbance | 24.5 | **< 0.001** | 37.7 | **< 0.001** | 2.8 | 0.103 | 8.8 | **0.005** | 38.4 | **< 0.001** | 23.7 | **< 0.001** |
| Hab × Fert | 1.3 | 0.267 | 0.2 | 0.662 | 0.8 | 0.374 | 1.4 | 0.249 | 0.1 | 0.777 | 0.0 | 0.834 |
| Hab × Dist | 1.2 | 0.287 | 6.5 | **0.014** | 1.8 | 0.189 | 1.1 | 0.313 | 4.0 | 0.051 | 1.7 | 0.203 |
| Fert × Dist | 1.9 | 0.174 | 0.1 | 0.719 | 1.5 | 0.233 | 9.9 | **0.003** | 0.0 | 0.911 | 2.6 | 0.117 |
| Hab × Fert × Dist | 0.8 | 0.366 | 0.0 | 0.861 | 3.5 | 0.067 | 0.8 | 0.389 | 0.0 | 0.878 | 0.8 | 0.390 |

*Logarithmic transformations were used to meet the requirements of the linear mixed model.

**Supplementary Table 8.** Mean concentrations of soil and microbial N (mg kg^-1^ SOM) and C (mg g^-1^ SOM). Values are mean and S.E. in parentheses, N = 6 in the mountain birch forest, N = 7 in the tundra heath. Values are mean + S.E. in parentheses.

|  | Mountain birch forest | | | | Tundra | | | |
| --- | --- | --- | --- | --- | --- | --- | --- | --- |
|  | Control | N-fertilized | Disturbed | N-fertilized and Disturbed | Control | N-fertilized | Disturbed | N-fertilized and Disturbed |
| NH_4_-N | 19.0 (9.8) | 12.7 (2.4) | 30.5 (5.2) | 22.9 (3.1) | 8.9 (1.5) | 22.5 (8.6) | 43.5 (11.6) | 32.2 (5.1) |
| NO_3_-N | 8.1 (0.6) | 8.4 (1.4) | 20.2 (4.1) | 14.8 (2.4) | 8.8 (5.3) | 5.9 (1.4) | 32.6 (9.3) | 34.2 (12.3) |
| Org N | 134.0 (31.3) | 109.4 (22.2) | 27.8 (12.1) | 173.4 (54.1) | 93.0 (39.9) | 110.0 (31.2) | 98.6 (62.1) | 80.8 (37.2) |
| Microbial N | 583.8 (58.3) | 585.3 (53.8) | 892.3 (128.1) | 477.5 (63.6) | 412.1 (23.1) | 444.7 (31.3) | 736.3 (116.0) | 532.8 (62.8) |
| DOC | 1.95 (0.17) | 1.81 (0.24) | 3.25 (0.52) | 3.07 (0.47) | 1.35 (0.21) | 1.29 (0.17) | 3.78 (0.63) | 3.87 (0.61) |
| Microbial C | 5.96 (0.19) | 5.76 (0.40) | 9.2 (1.46) | 7.89 (0.95) | 4.48 (0.49) | 5.26 (1.01) | 10.96 (2.01) | 8.03 (0.51) |
|  |  |  |  |  |  |  |  |  |

**Supplementary Table 9.** Correlations (r^2^) of soil properties and N and C pools with the NMDS-ordination pattern describing old disturbance and N-fertilization treatments conducted in 2002, in 2020. Significance of correlations (P) is based on 999 permutations. Signif. codes: 0 '***' 0.001 '**' 0.01 '*' 0.05 '.' 0.1 ' ' 1

|  | r^2^ | Pr(>r) |
| --- | --- | --- |
|  |  |  |
| OMC% | 0.4904 | 0.001 *** |
| SOM stock | 0.4671 | 0.001 *** |
| Soil pH | 0.3225 | 0.001 *** |
| Moisture % | 0.4799 | 0.001 *** |
|  |  |  |
| **A. Per Soil Organic Matter** |  |  |
| NH_4_-N | 0.4257 | 0.001 *** |
| NO_3_-N | 0.3909 | 0.001 *** |
| Organic N | 0.0006 | 0.984 |
| Microbial N | 0.0770 | 0.136 |
| DOC | 0.3754 | 0.001 *** |
| Microbial C | 0.3517 | 0.001 *** |
| 1. **Per Area** |  |  |
| NH_4_-N | 0.0299 | 0.439 |
| NO_3_-N | 0.1188 | 0.054 |
| Organic N | 0.0669 | 0.160 |
| Microbial N | 0.3239 | 0.001 *** |
| DOC | 0.1532 | 0.011 * |
| Microbial C | 0.3472 | 0.001 *** |
|  |  |  |
